# Vaccination and Smallpox

**Published:** 1921-11

**Authors:** 


### VACCINATION AND SMALL-POX.

### MEMORANDUM BY THE MINISTRY OF HEALTH.

The Ministry of Health has recently published a report on small-pox and vaccination as No. 8 in its reports on public health and medical subjects. Our readers are aware of the general excellence of this series of reports by the Ministry, and the present number on the value of vaccination is of the same standard of merit.

We give below a few extracts from the report.

### Epidemic Small-pox : Variations in Type.

Small-pox is a disease that recurs in epidemic form from time to time, and during these epidemic outbursts it spreads rapidly over wide surfaces of the world and its infectiousness is largely increased. Although the infectivity of the disease is enhanced, the virulence of the infection is not always increased, so that in one epidemic the proportion of fatal cases to the total number of known cases of small-pox may be very high, while in another epidemic the proportion of deaths to cases may be small. That the severity of the disease might vary in different epidemics within very wide limits was well known to early writers on small-pox; and at the present time more than one type of the disease exists. On the American continent, although severe cases of small-pox occur, the prevailing type is mild and the fatal cases are few. In a recent introduction of this type of disease into England some ninety cases occurred without a single death. In the East the type is severe, and when imported into this country the disease maintains this character.

### Decrease in Small-pox Mortality.

The first quarter of the nineteenth century was characterised in this and in other countries by a striking decrease of small-pox mortality. It must be borne in mind that at the time of the introduction of vaccination a large proportion of the population was protected by previous attacks of small-pox, either natural or inoculated, and that the amount of vaccination adequate to afford a great protection in the earlier years of the century ceased to be adequate for the later years, as those who had suffered attack by small-pox were removed from the population in the natural course of events by death.

### The Influence of Improved Sanitation.

It is reasonable to suppose that overcrowding of houses on land and of persons in houses, and that uncleanliness of persons and of their surroundings, would favour the spread of infection from person to person; and the argument has been advanced that improved sanitary conditions would tend to lessen the fatal results, and that in consequence the mortality from small-pox would be correspondingly lowered. Compared with 100 years ago, the country has purer water supplies and better sewerage systems, various Public Health Acts have been passed under which medical officers of health and sanitary inspectors have been appointed, the lighting and ventilation of dwellings have been improved, and power has been given to local sanitary authorities to deal with nuisances, and it is now more generally recognised that certain diseases are infectious. Other changes, however, have been in operation. There are greater facilities for travelling and greater interchange of persons between districts, resulting in increased opportunities for spread of infection.

But when small-pox breaks out, it visits with strict impartiality healthy and unhealthy districts, and the incidence of attack and death falls on rich and poor alike, subject only to one condition—the absence or the presence of the protection afforded by vaccination.

Concerning the recent state of vaccination among the population of England and Wales, the report states, "At the present time a considerable proportion of the adult male population is protected, owing to having been revaccinated while serving in the Navy and Army during the war. The adult female population is protected to a lesser extent, although many women who served in the auxiliary corps were revaccinated."

### The Ministry's Summary.

We know that the mortality from small-pox is much less now than in pre-vaccination times, that the greatest diminution in the small-pox mortality is found in the early years of life in which there is most vaccination; that in countries in which there is much vaccination and re-vaccination relatively to the population, there is little small-pox; that in places where small-pox prevails it attacks a much greater proportion of the unvaccinated than the vaccinated, especially where the vaccinations are comparatively recent; that in houses invaded by small-pox in the course of an outbreak not nearly so many of the vaccinated inmates are attacked as of the unvaccinated in proportion to their numbers; that the fatality rate among persons attacked by small-pox is much greater age for age among the unvaccinated than among the vaccinated; that the degree of protection conferred by vaccination corresponds to the thoroughness with which the operation has been performed, four marks affording much better protection than one or two; that the protection afforded by vaccination wanes with lapse of time; that improved sanitation, however beneficial in itself, cannot account for these facts; and that though early diagnosis, prompt isolation of small-pox patients in suitable hospitals, effective disinfection, supervision of "contacts," and other such public health measures are invaluable, they are no substitute for vaccination.

The report is made complete by the inclusion of several tables of statistics. This series of reports on public health and medical subjects is published by H.M. Stationary Office, and the present report can be obtained for the sum of threepence. We think that its wide circulation in those areas which are threatened with an epidemic of small-pox would be of great value in educating the public, and we welcome, therefore, the fact that the price of this pamphlet is so moderate.

---

The Ministry of Health has issued to sanitary authorities throughout the country a circular on the subject of anthrax from shaving-brushes, stating:

"With regard to shaving-brushes made in this country, the Minister is advised that, as the hair in brushes after manufacture cannot be effectively sterilised, it is essential for the protection of the public health that all practicable measures should be taken to ensure that the hair used for making shaving-brushes is efficiently disinfected before the brushes are manufactured, and he would accordingly draw attention to methods of disinfection of hair."

The circular concludes: "The Minister considers that there would be great advantage if local authorities possessing efficient steam disinfecting apparatus would afford brush manufacturers in their districts all the facilities they require for the disinfection of hair, upon terms to be mutually agreed upon."

